# HIV Associated Preeclampsia: A Multifactorial Appraisal

**DOI:** 10.3390/ijms22179157

**Published:** 2021-08-25

**Authors:** Thajasvarie Naicker, Nalini Govender, Tashlen Abel, Nitalia Naidoo, Merantha Moodley, Yazira Pillay, Shoohana Singh, Olive Pearl Khaliq, Jagidesa Moodley

**Affiliations:** 1 Optics & Imaging Centre, Doris Duke Medical Research Institute, College of Health Sciences, University of KwaZulu-Natal, Durban 4041, South Africa; yazira123@gmail.com (Y.P.); Singhs5@ukzn.ac.za (S.S.); 2 Department of Basic Medical Sciences, Faculty of Health Sciences, Durban University of Technology, Durban 4041, South Africa; nalinip@dut.ac.za; 3 Women’s Health and HIV Research Group, Department of Obstetrics and Gynaecology, School of Clinical Medicine, College of Health Sciences, University of KwaZulu-Natal, Durban 4041, South Africa; tashlen.abel@gmail.com (T.A.); nitaliatally@gmail.com (N.N.); meran1204@gmail.com (M.M.); saminah.khaliq@gmail.com (O.P.K.); jmog@ukzn.ac.za (J.M.)

**Keywords:** HIV, antiretroviral therapy, preeclampsia, complement system, angiogenesis, RAAS, Netosis

## Abstract

*Introduction:* This review explores angiogenesis, vascular dysfunction, the complement system, RAAS, apoptosis and NETosis as potential pathways that are dysregulated during preeclampsia, HIV infection and ART usage. *Results:* HIV-1 accessory and matrix proteins are protagonists for the elevation of oxidative stress, apoptosis, angiogenesis, and elevation of adhesion markers. Despite the immunodeficiency during HIV-1 infection, HIV-1 exploits our cellular defence arsenal by escaping cell-mediated lysis, yet HIV-1 infectivity is enhanced via C5a release of TNF-α and IL-6. This review demonstrates that PE is an oxidatively stressed microenvironment associated with increased apoptosis and NETosis, but with a decline in angiogenesis. Immune reconstitution in the duality of HIV-1 and PE by protease inhibitors, HAART and nucleoside reverse transcriptase, affect similar cellular pathways that eventuate in loss of endothelial cell integrity and, hence, its dysfunction. *Conclusions:* HIV-1 infection, preeclampsia and ARTs differentially affect endothelial cell function. In the synergy of both conditions, endothelial dysfunction predominates. This knowledge will help us to understand the effect of HIV infection and ART on immune reconstitution in preeclampsia.

## 1. Introduction

Low and middle-income countries have higher maternal mortality rates (462 per 100,000 live births) compared to high-income countries (11 per 100,000 live births) [1]. A systematic analysis conducted by the World Health Organisation (WHO) indicates that haemorrhage and hypertensive disorders of pregnancy are the major causes of direct maternal deaths worldwide [1,2]. Preeclampsia (PE) is a hypertensive disorder of pregnancy and one of the main direct causes of maternal morbidity and mortality, whereas human immunodeficiency virus (HIV) infection in pregnancy remains the leading indirect cause of maternal deaths [3].

Women of reproductive age account for 52% of all adult HIV infections worldwide [1,4]. In poor income countries, HIV infected women have 5.2–8 times the risk of direct maternal mortality compared to HIV uninfected women [5,6]. One in three pregnant women attending antenatal care in South Africa is HIV infected [7,8]. Despite antiretroviral therapy (ART) improving life expectancy and reducing mother to child HIV transmission, a significant number of maternal mortality rates from HIV infection still prevail [9,10]. In addition, adverse pregnancy outcomes, low birthweight and preterm births are reported in individuals receiving ART [11].

The COVID-19 pandemic caused by the severe acute respiratory syndrome coronavirus 2 (SARS-CoV-2) is also associated with an increased risk of severe outcomes in pregnancy than the general population [12,13,14]. The SARS-CoV-2 infection superimposed on HIV-associated pregnancy is complex as both impact the inflammatory response and influence endothelial function [15,16].

The idiopathic condition of PE is diagnosed by the presence of de novo hypertension after 20 weeks of gestation with/without proteinuria and/or evidence of maternal acute kidney injury, liver dysfunction, neurological features, haemolysis or thrombocytopenia, or foetal growth restriction [17]. The pathogenesis of PE remains unknown; however, it involves defective placentation and inadequate spiral artery transformation in early pregnancy, resulting in reduced blood flow and inadequate nutrients and oxygen supply to the neonate [18]. The consequent hypoxic conditions promote widespread oxidative stress, angiogenic imbalance and an amplified immune and inflammatory response [19]. Endothelial dysfunction induces the maternal syndrome of PE [16].

## 2. Immune Response and the Complement System

In a healthy pregnancy, several immunological adaptations support immunologic tolerance to the semi-allogenic foetus and placenta and simultaneously protects the mother and foetus against pathogens [20]. The inhibitory action of maternal immune cells to paternal antigens in the developing foetus is key to a successful pregnancy. The evolving theory encompassing immune modulation during pregnancy suggests that placentation synergistically develops with the immune system to capitalize on the crosstalk linking trophoblast and immune cells, instead of circumventing the maternal decidual immune responses [21]. Maternal decidual natural killer cells (dNK) and foetal trophoblasts confer immunity at the maternal-foetal interface via receptor-signalling. Immune tolerance occurs via human leukocyte antigens (HLA), albeit their absence on syncytiotrophoblasts precludes them from detecting paternal antigens [21]. Stable forms of HLA-E and HLA-G are expressed by the human foetal extravillous trophoblasts (EVT), whilst HLA-C allotypes express an affinity to either stimulatory or inhibitory maternal dNK receptors [21].

A breakdown of immune tolerance or immunological incompatibility between the mother and foetus may be associated with PE development [22]. Preeclampsia is dominated by a proinflammatory (Th1) response [23]; however, in combination with HIV infection and usage of HAART, the Th1 response is exacerbated [24]. Placental perfusion and invasion are enhanced by the stimulatory effects of foetal HLA-C genetic variants, in contrast to their inhibitory invasive effects in PE [21].

The complement system, a part of the innate immunity, is associated with the host defence and removal of apoptotic cells, injured tissue debris and immune complexes, and mediates one’s adaptive response [25]. Activation of the complement cascade results in inflammation, opsonisation and lysis by forming the membrane attack complex [25,26].

In pregnancy, a degree of complement activation is required during placentation for development, and to support the removal of placental apoptotic debris [22,27]. Foetal protection at the maternal-foetal interface is enhanced via complement regulators [27]. Excessive activation or dysregulation, however, can result in tissue damage, anaphylatoxin release with consequent inflammation, vascular leakage and thrombosis [28]. Previous studies demonstrate a dysregulation of the circulatory and placental complement system in PE [29,30,31,32], suggestive that a fine balance must be maintained between complement activation and regulation.

HIV-1 infection causes immunodeficiency, hyperactivation of the immune system and chronic inflammation [33]. Nonetheless, activation of the complement system is an essential arsenal in the defence against HIV; however, it may also enhance HIV-1 infectivity [34]. The membrane spikes of HIV-1 consist of 3 glycoprotein (gp) 120 molecules linked together and anchored to the transmembrane protein, gp41. The glycoprotein 41 binds to complement component 1q (C1q) and activates the complement classical pathway, whereas the HIV-1 envelope gp120 binds to mannose-binding lectins and neutralises the virus [35]. This action triggers the activation of the complement lectin pathway, preventing viral entry [36]. Nonetheless, C5a increases TNF-α and IL-6 release, thereby promoting HIV-1 infection, whilst inhibition of its receptor C5aR reverses this action [37].

Additionally, HIV-1 incorporates the host cell complement regulatory proteins (CD59 and CD55) on its viral envelope to escape complement-mediated lysis [35]. The subsequent complement opsonized HIV-1 enhances viral infection of T and B cell lines [38]. Both HIV and PE are characterised by chronic inflammation, which may be aggravated by excessive complement activation [39,40].

People living with HIV and receiving ART still encounter a high rate of non-AIDS related comorbidities, which may be related to increased levels of systemic immune activation [40,41]. A 54% increase in complement activation in ART-treated adults versus healthy controls has been observed [40]. A recent study revealed a significant elevation in several complement factors in ART-treated HIV infected individuals vs. seronegative controls [41]. In addition, an upregulation in complement regulatory proteins CD35 and CD55 was demonstrated in ART-treated HIV infected PE compared to normotensive pregnant women [42], suggesting that HIV confers a protective effect in PE. All pregnant HIV infected women were on ART [42]. Furthermore, activation of the complement system may lead to endothelial cell injury and subsequent activation of the clotting cascade [43].

## 3. HIV Infection and Endothelial Dysfunction

**Figure 1:**
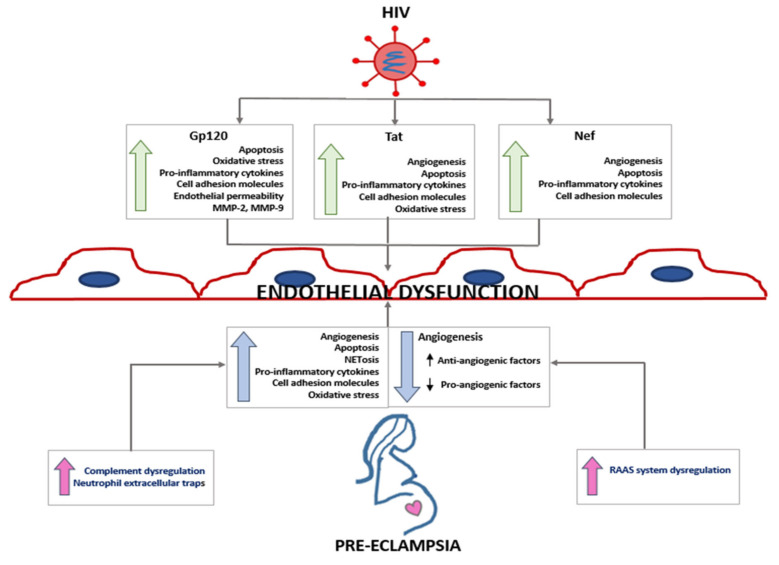
The homology between HIV-1 and PE response to endothelial dysfunction. HIV-1 accessory proteins gp120, tat and nef, as well as matrix proteins, are protagonists of the cellular machinery that culminate in endothelial cell dysfunction. Adapted from [30].

Similar to PE [44], HIV infected individuals display increased systemic oxidative stress because it is able to suppress endogenous antioxidant enzymatic mechanisms [45,46]. In addition, syncytin, a captured retroviral gene, is expressed by placental syncytiotrophoblasts [47]. The dysregulation of this viral fusion protein of endosymbiotic endogenous retroviruses may be implicated in PE pathogenesis [47]. Recent studies suggest a causative role of the HIV-1-proteins tat, gp120 and nef on endothelial dysfunction (Figure 1) [48].

The endothelium plays an important role in the pathogenesis of viral infection [33]. In HIV infection, endothelial cell (EC) injury and loss of its integrity is due to changes in blood flow and increased circulation of molecules such as IL-6, D-dimer, fibrinogen, C-reactive protein, TNF-α, soluble intercellular adhesion molecule (sICAM), soluble vascular cell adhesion molecule (sVCAM) and endothelial microvesicles [49]. The HIV-1 tat protein is a powerful angiogenic factor, and it binds to cell adhesion molecules (integrins αvβ5, α5β1 and αvβ3, fibronectin and vitronectin) [50]. It also binds to Flk-1/KDR, FLt-1 and MAP kinase, thereby influencing EC growth, invasion and angiogenesis [33,51]. The Tat protein is released from infected cells and causes endothelial cell dysfunction (Figure 1) even when the patient is on ART [52].

The HIV-1 nef protein moves into endothelial cells via nanotube-like conduits mediated by a cytoskeletal rearrangement that affects their permeability and reduces tight junction proteins [53,54,55]. Additionally, nef induces apoptosis via the mitochondrial and Fas/FasL pathways and decreases nitric oxide production and promotes ROS production and consequent oxidative stress and apoptosis [56].

The accessory protein gp120 promotes apoptosis and the production of ET-1, reducing the nitric oxide (NO) synthesized by the NO synthase, hence promoting EC dysfunction [57]. Glycoprotein 120 mediates apoptosis via CXCR4-dependent caspase and p38 MAPK promotion [58]. Post EC injury, an elevated expression of the potent vasoconstrictor ET-1, occurs [57,59]. The HIV-1 matrix protein, p17, interacts with CXCR1 and CXCR2 on endothelial cells to activate the MAPK/ERK and PI3K/Akt signalling pathways, thereby prompting angiogenesis and lymphangiogenesis [60].

## 4. Vascular Endothelial Growth Factors: Pivotal Angiogenesis Regulators

**Figure 2:**
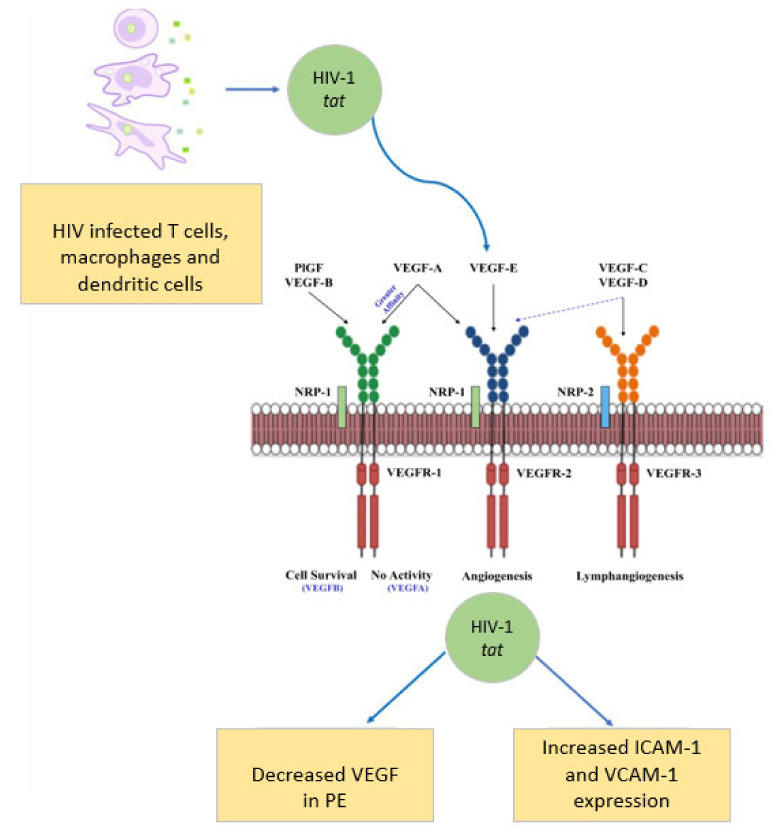
HIV-1 accessory protein Tat is released from infected cells, decreases bioavailability of VEGF and increases cell adhesion expression, thereby promoting endothelial cell dysfunction. Adapted from [67,68].

The vascular endothelial growth factor (VEGF) family are potent regulators of the angiogenic signalling and includes VEGF-A, VEGF-B, VEGF-C, VEGF-D and placental growth factor (PlGF), as well as VEGF receptors: (VEGFR) VEGFR-1, VEGFR-2 and VEGFR-3 [61,62,63]. Specifically, VEGFs regulate cell proliferation, invasion and migration during angiogenesis [64]. Signal transduction of VEGF-A occurs via VEGFR-1 and VEGFR-2, whilst VEGF-B and PlGF activate VEGFR-1 [65], and VEGF-C and VEGF-D show an affinity for VEGFR-3 (Figure 2) [66].

## 5. Angiogenic Imbalance in Preeclampsia

Several studies have reported a decrease of VEGF-A in maternal circulation with a concomitant increase of soluble fms-like tyrosine kinase (sFlt-1), the soluble form of VEGFR-1 [69]. Gene expression profiling confirms an upregulation of sFlt-1 mRNA in preeclamptic placentae [70]. Notably, an elevation in sFlt-1 and sEng was observed in maternal circulation almost five weeks prior to the onset of PE development [69,70,71,72]. Excess sFlt-1 in PE pregnancies reduces the circulating levels of VEGF and PlGF and antagonises their biological function [71,73], resulting in endothelial dysfunction [74,75]. In view of this, the sFlt-1/PlGF ratio is a highly recommended clinical tool for differentiating between diverse pregnancy-related hypertensive syndromes [76,77,78,79]. Furthermore, soluble endoglin (sEng) and sFlt-1 exert inhibitory effects on VEGF, PlGF and TGF-β signalling, thus activating the endothelial nitric oxide synthase (eNOS) system and vasomotor effects [80]. Similar to sFlt-1, elevated serum sEng levels diminish circulating TGF-β levels and disrupt subsequent signalling [81].

Moreover, our group demonstrated increased circulating levels of sFlt-1 and sEng in PE compared to normotensive pregnancies, irrespective of the HIV status, albeit with a downward trend of circulating sFlt-1 and sEng in the HIV-infected vs. HIV uninfected groups [82,83]. HIV-infected pregnant women develop PE at lower rates compared to HIV uninfected women [8]; however, their risk of PE development is debatable [84]. Govender et al. reported no significant difference between sFlt-1, sEng and PlGF levels in HIV-associated PE [83]. An overview of the role of these angiogenic and antiangiogenic factors in PE development is illustrated in Table 1.

Our group also demonstrated the role of various other angiogenic factors in PE development [16,85,86,87,88,89,90]. We have shown an elevation of TN-C [86], Ang-2 and Eng [87], sVEGFR-1 with concurrent reduction in PECAM-1 and sVEGFR-2 levels [88] and a downregulation in plasma NF-κB and IκB-α expression [90] in PE versus normotensive pregnancies, regardless of HIV status, indicative of their potential role in PE development. However, HIV status did not affect the expression of sTie2 and sHER2 [85] nor sE-selectin [16] in our cohorts, regardless of pregnancy type, which is suggestive that HAART reconstitutes the immune system.

**Table 1:** Angiogenic and antiangiogenic markers in Preeclampsia development.

Angiogenic/ Antiangiogenic/ Lymphangiogenic Factors	Main Findings	References
VEGFR-2	Downregulation of VEGFR-2 in PE compared to normotensive. Downregulation of VEGF-dependent cell proliferation when the VEGFR-2 signalling pathway was blocked in mice.	[70,91,92,93]
VEGFR-3	Decidual VEGFR-3 is significantly downregulated in PE placentae vs. normotensive; indicative of lymphangiogenic inhibition on PE.	[94,95]
VEGF-A	Reduced VEGF-A expression in PE compared to normotensive pregnancies. Sequestering and binding of VEGF-A by its soluble receptors.	[71,96,97,98]
VEGF-C	Significant increase in VEGF-C expression in preeclampsia compared to normotensives; the pro-lymphangiogenic state evident in PE is linked with oedema and hypertension. No dysregulation in VEGF-C expression noted in HIV infected preeclamptic women vs. uninfected women; may be due to immune reconstitution in response to ARV use.	[99,100]
PlGF	Downregulation in PlGF levels noted in PE compared to normotensive pregnancies. Greater PlGF reduction seen in EOPE, due to binding and sequestering of PlGF by the soluble VEGF receptors.	[70,71,96,98]
sFlt-1	Circulating sFlt-1 levels upregulated in preeclampsia prior to delivery and is reduced to baseline 48–72 h postdelivery. High sFlt-1 levels correlate inversely with decreased free PlGF levels during preeclampsia. Circulating PlGF levels are downregulated in PE and correlated elevation in total VEGF-A with soluble VEGFR-1. Membrane-bound VEGFR1 is reduced in the placental bed and correlates with poor uteroplacental progression. Upregulation of new sFlt-1 isoforms with differing mRNA lengths in preeclamptic placentas. Circulating sFlt-1-14 identified as a major VEGF inhibitor in PE.	[71,96,101,102,103,104]
sEng	Upregulation of sEng in confirmed preeclampsia and in pregnancies prior to symptom onset. Midpregnancy urinary PlGF levels correlates with the consequent preterm PE development. Thrombocytopenia, haemolysis and HELLP was noted as a severe PE outcome in animals treated with both sFLT1 and sEng. sEng upregulated in PE patients with severe complications including placental abruption, HELLP syndrome, eclampsia and IUGR.	[69,70,73,80,105,106,107,108,109]

Our research group has recently established that, in PE, a pre-delivery sFlt-1/PIGF proportion (<181.5) is an encouraging predictor value for excluding the need for >3 slow-acting and/or a rapid-acting antihypertensive drug management in the immediate postpartum period [89]. To our knowledge, we are the first group to provide the cut-off thresholds of pre-delivery sFlt-1/PIGF ratio that can be used to foresee antihypertensive use in the postpartum period in PE and normotensive pregnancy. These results implicate the usefulness of angiogenic factors in examining the manifestation and progression of PE development. Future prospective studies using larger sample sizes that encompass all three trimesters will assist in determining the clinical usefulness of these biomarkers in advancing the clinical management strategies for PE.

## 6. Angiogenesis and Lymphangiogenesis

Neuropilin-1 (NRP-1), a co-receptor for VEGFR-1 [61], binds to VEGF-2 in the presence of VEGF_165_, which stimulates phosphatidylinositol 3-kinase (PI3K) and activate protein kinase B (Akt) to promote angiogenesis [110,111]. Activation of VEGFR-3 enables lymphangiogenic regulation, which is also regulated by neuropilin-2 (NRP-2) [65,112]. Moreover, VEGF-A and PlGF binds to NRP-1 and enhances its affinity for VEGFR-2 [113]. A disruption to the equilibrium of these factors will inevitably result in an imbalance in angiogenesis and lymphangiogenesis. Thus, PE may be molecularly defined by a reduction in sVEGFR-3, decreased sVEGFR-2 and increased VEGF-C; however, the involvement of VEGF-B, VEGF-C and VEGF-D in PE development remains debatable.

The immunoexpression of NRP-1 is decreased in the syncytiotrophoblasts in PE compared to normotensive pregnancies, implicating NRP-1 in PE development [114]. Likewise, significantly lower NRP-1 and VEGF levels were observed in both PE and in homocysteine-induced PE mice, which is indicative of endothelial injury and consequent PE development [115]. Moreover, the downregulation of placental NRP-1 expression in foetal growth-restricted pregnancies complicated with absent end-diastolic flow in the umbilical artery correlates with PE development [116]. However, a lack of noticeable differences in NRP-1 and NRP-2 mRNA expression was noted between preeclamptic and normotensive human placentas [117]. Notably, NRP-1 was recently implicated in binding SARS-CoV-2 and showed direct involvement in SARS-CoV-2 entry by potentiating increased viral internalization and infectivity [118]. Investigations linked to NRP-1 and NRP-2 in HIV infection and PE in the HIV-COVID-19 pandemic thus requires urgent and intensive scrutiny.

## 7. Antiretroviral Therapy and Preeclampsia

The higher prevalence and mortality rates observed among PE cases are associated with ART use in comparison to HIV infection alone [16]. In the event of HIV infection, a pregnant women’s adherence to ART increases the risk of pre-term birth [119,120]. Additionally, hyper-vascularity of the placental terminal villi has been documented as a compensatory response to maternal ARV therapy [119]. Further large-scale studies are urgently required to investigate the effect of the duration of highly active antiretroviral treatment/therapy (HAART) on PE development.

Dysregulated angiogenesis, inflammation and oxidative/nitrosative stress resulting from placental maladaptation promote widespread endothelial dysfunction in PE. HIV accessory and matrix proteins also upregulate these pathways, which intensify endothelial injury. A summary of the impact of ART on various indicators of endothelial dysfunction is shown in Table 2.

## 8. Renin-Angiotensin-Aldosterone System in HIV Associated Preeclamptic Women on ART

The Renin-Angiotensin-Aldosterone System (RAAS) depends on several receptors for signal transduction of the vasoconstrictor angiotensin II (ANGII), which is liable for increasing blood pressure in PE. Irregular renin-angiotensin stimulation may be linked to the pathophysiology of hypertension and insulin resistance in HIV infection [131], with elevated plasma renin activity resulting from increased RAAS upstream activation [132]. This anomaly is elaborated by the similarity between the structure of HIV-1 protease and that of renin [133]. An increased renin production in immune cells occurs due to the presence of HIV, thereby activating RAAS. Furthermore, renin contributes to HIV replication via the renin signalling cascade and cleavage of the HIV Gag polyproteins [131].

Bouba et al., (2003) reported a significant association between circulating angiotensin (AGT) gene polymorphisms (M235T) and PE [134]. Similarly, Aung et al. (2017) reported a significant increase in the AGT variant (M235T) in PE compared to controls [135]. In contrast to AGT gene polymorphisms, the ATR1 variant (A1166C) showed no association with PE, despite the upregulation reported in both the circulation and the placentae of women with PE compared to controls [135,136]. However, ATR2 (C4599A) showed a significant association with PE in women with a BMI of ≥25 kg/m^2^ [132]. Moreover, a significant reduction of AT4R in the circulation and the placentae of women with PE was reported [136,137,138], albeit a non-significant association of genetic variant (rs18059) with the pathogenesis of PE [139].

The association of HIV treatment (HAART, protease inhibitors and/or nucleoside reverse transcriptase inhibitors) and RAAS is currently unclear. However, evidence shows that HIV treatment increases the risk of hypertension. A study conducted in Cameroon that was comprised of HIV infected non-pregnant patients on treatment reported the development of hypertension in 36.4% of the population, in comparison to 13.3% HIV positive patients without treatment [140]. Furthermore, the development of PE has been observed in patients on HAART [141,142]. Reports suggest that HIV infected pregnant women are protected against PE but develop the disease on ART initiation [142]. The precise pathogenesis underlying this mechanism and the effect of HAART on RAAS gene expression and RAAS genetic variants is poorly understood and thus requires further investigation. Notably, SARS-CoV-2 infection exploits ACE2 to induce endothelial dysfunction and hypertension, thereby mimicking angiotensin II-mediated PE in severe cases of infection [143]. Upregulated ACE2 in pregnancy is a possible risk factor for SARS-CoV-2 infection and subsequent PE development [16].

## 9. Cell Death-Neutrophil Extracellular Traps (NETs): Immune Defence

Several studies have implicated a dysregulation of apoptosis on PE development [144,145]. The former study demonstrates increased trophoblast apoptosis in the placental bed of preeclamptic compared to normotensive pregnancies with a concurrent absence of proliferation at term; this enhancement interferes with the process of placentation and eventuates in systemic endothelial damage. In contrast to apoptosis, NETosis is the formation of neutrophil extracellular traps (NETs), a novel neutrophil cell death mechanism [146]. It is triggered in response to pathogenic and pro-inflammatory stimuli, which activates neutrophils to expel their intra-nuclear and intra-cytoplasmic contents in NETs [147]. These NETs are a sticky web of chromatic decorated with histones and granules to trap invading pathogens or pro-inflammatory molecules before eliciting cell death [146,147,148,149]. A hyper-activation or defective homeostatic clearance of NETs result in various adverse outcomes such as SARS-CoV-2, rheumatoid arthritis and PE [150,151,152,153].

## 10. NETs in HIV-Associated Preeclampsia

In normotensive pregnancies, NETs are expressed in the placental decidua within the conducting villi circulation, surrounding the conducting and exchange villi in the intervillous space [153,154]. In PE, syncytiotrophoblasts (STB) release elevated levels of pro-inflammatory microparticles and interleukin-8 (IL-8), which activate neutrophils to undergo excessive NETosis [153,154]. These NETs contribute to the hyper-inflammatory state observed in PE and exacerbate the underlying PE-compromised endothelial dysfunction [155]. Evidently, elevated levels of maternal cell-free DNA correlate with the severity of PE [156] and further contributes to placental malfunction and foetal rejection by autoimmune activation [157]. NETs are elevated in HIV infection [154,158] and function in trapping and inactivating the virions at a transcriptional level [158,159]. However, as a viral evasion mechanism, HIV stimulates the release of anti-inflammatory IL-10 from dendritic cells to suppress the levels of NETs [158]. Because NETs are individually elevated in PE and HIV infections, it is surprisingly evident that NETs are suppressed in the co-infection of HIV-associated PE; however, this suppression may be attributed to ART usage [154]. This synergy is plausibly attributed to the enhanced expression of dendritic cells in PE [160]—serving as a reservoir for HIV-mediated NETs suppression [154].

## 11. Recommendations and Therapy Guidelines: HIV Infection and Preeclampsia

In view of the scarcity of available data on drug-to-drug inventions in HIV associated pregnancy, current recommendations for the use of anti-hypertensive drugs in PE are valid. There is no long-term data on the use of anti-hypertensive drugs in HIV infected patients receiving HAART, therefore clinical practice should be based on a case by case scenario. If the hypertension is severe, then anti-hypertensive agents must be used to lower the immediate threat to the mother and baby. Viral replication may be controlled by an antibody response without HAART in pregnancy [161]; however, pregnancy itself does weaken the immune system. Therefore, it is not advisable to temporarily stop ARV usage in pregnant women to prevent PE development as the immune system may not be strong enough to fight viral replication. Further studies in a pregnant cohort are required to investigate antibody neutralization without ARV usage.

From a clinical perspective, it is advisable that patients with HIV infection evaluate their viral load prior to planning a pregnancy, as viral load is not an indication for termination of pregnancy. There is a paucity of data on pregnancy that compares the efficacy of anti-hypertensive drugs in pregnancy; however, Labetalol, an alpha beta blocker may be an appropriate choice in treating HIV-infected PE patients. Notably, no data is currently available to suggest that PE increases the risk of maternal-foetal HIV transmission.

## 12. Conclusions

This review explores research evidence that may aid in reducing the main direct (preeclampsia) and indirect (HIV infection) causes of global maternal mortality. Both Preeclampsia and HIV infection have opposing immune responses; however, this is offset by the immune reconstitution that occurs with ART usage. Moreover, both conditions culminate in endothelial cell dysfunction (Figure 1). The accessory and matrix proteins of HIV-1 differentially contribute to elevated oxidative stress, apoptosis and the secretion of pro-inflammatory cytokines, cell adhesion molecules and angiogenesis. These events also occur in the hypoxic microenvironment of PE; hence, in the synergy of HIV associated PE, there is an exacerbation of these events, thereby affecting downstream targets. It is possible that HIV-1 has developed mechanisms to escape complement-mediated lysis whilst simultaneously increasing its infectivity of host cells, thereby disturbing foetal defence at the maternal-foetal interface.

## Figures and Tables

**Table 2:** Antiretroviral Therapy and HIV associated PE.

Antiretroviral Type	Main Findings	References
Nucleoside/nucleotide reverse transcriptase inhibitors	Immune reconstitution Decreased endothelial cell proliferation, migration Elevated mitochondrial oxidative stress Defective tyrosine kinase receptor and VEGFR-2 signalling Increased reactive oxygen species (ROS) generation in trophoblast apoptosis predisposing PE and/or IUGR	[121,122]
Protease inhibitors	Immune reconstitution preluding PE development Decreased progesterone in trophoblast cells impede invasion Dysregulated uterine decidualization and spiral artery remodelling Decreased VEGF, placental growth factor (PlGF), angiopoietin-2, interferon-gamma and matrix metalloproteinase 9 (MMP-9) in decidual cell Uterine natural killer (uNK) cell depletion Incomplete trophoblast cell invasion following decreased expression of the transcription factor STAT3	[123,124,125]
HAART	Immune reconstitution Dysregulated nuclear factor kappa B (NF-κB) transcription factors and, hence, decreased MMP and VEGF expression Increased sFlt-1 and sEng Decreased PlGF and VCAM-1	[83,88,126,127,128,129,130]

## Data Availability

Not applicable.
